# Board of Naval Surgeons

**Published:** 1845-01

**Authors:** 


*Naval Surgeons.*—The Board of Naval Surgeons which was convened in Philadelphia on the 7th of October last, and closed its proceedings on the 25th ultimo, reported the following Assistant Surgeons as having been examined and found qualified for promotion:

*Of the date of June*, 1838.—William B. Sinclair and Stephen A. McCeery.

